# Capsular serotype and antibiotic resistance of group B streptococci isolated from pregnant women in Ardabil, Iran

**Published:** 2012-09

**Authors:** E Jannati, M Roshani, M Arzanlou, S Habibzadeh, G Rahimi, R Shapuri

**Affiliations:** 1 Department of Microbiology, School of Sciences, Ardabil Branch, Islamic Azad University, Ardabil, Iran; 2 General practitioner, Ardabil University of Medical sciences, Ardabil, Iran; 3 Department of Microbiology, School of Medicine, Ardabil University of Medical Sciences, Ardabil, Iran; 4 Department of Infectious Diseases, School of Medicine, Ardabil University of Medical Sciences, Ardabil, Iran; 5 Department of Obstetrics and Gynecology, School of Medicine, Ardabil University of Medical Sciences, Ardabil, Iran; 6 Biology Research Center, Zanjan Branch, Islamic Azad University, Zanjan, Iran

**Keywords:** Antibiotic resistance, Group B Streptococci, Serotype

## Abstract

**Background and Objectives:**

Group B Streptococci (GBS) is a major cause of neonatal and maternal infections. The aim of this study was to determine the serotype distribution and antibiotic resistance profile of GBS strains isolated from pregnant women in Ardabil.

**Materials and Methods:**

Antibiotic resistance of 56 GBS isolates was investigated using E-test strips and disk-diffusion method. Serotyping was performed using capsular antiserum.

**Results:**

The results of MIC tests showed all isolates were susceptible to ampicillin, vancomycin and penicillin. One isolate (1.7%) showed reduced susceptibility pattern to penicillin (MIC; 0.25 µg/ml). There were 3 (5.3%) isolates semi-sensitive (0.25-1 µg/ml) to erythromycin (2; 0.5 µg/ml and 1; 0.38 µg/ml) and 2 (3.5%) isolates to clindamycin (1; 0.5 µg/ml, 1; 0.38 µg/ml). Additionally, 2 (3.5%) isolates were resistant to clindamycin (1; 16 µg/ml, 1; 2 µg/ml). According to the disk diffusion test, 47 (83.9%), 8 (14.2%) and 7 (12.5%) isolates were resistant to Co-trimoxazole, ciprofloxacin and ceftriaxone respectively. Serotypes V (19.6%), II (12.5%) and IV (12.5%) were the most frequent followed by serotypes III (10.7%) and VI (10.7%), Ib (8.9%), Ia (7/1%), VII (5/3%) and VIII (5/3%); 7.1% of strains were nontypeable.

**Conclusions:**

In this study, most isolates were sensitive to common antibiotics, but increased resistance to other antibiotics indicates the importance of monitoring of antibiotic resistance in group B streptococci over time.

## INTRODUCTION

Group B Streptococci (GBS) are the most frequent cause of severe, life-threatening neonatal infections (1). Significant infections are also seen in adult women as a complication of pregnancy and in the elderly and immunocompromised hosts (2). Commonly, 10–40 % of pregnant mothers carry GBS in their genitourinary and gastrointestinal tracts (3). Maternal colonization with GBS is the predominant risk factor for the development of invasive neonatal GBS disease. Early-onset neonatal infections (Sepsis, pneumonia and meningitis) generally result from vertical transmission of bacterium from asymptomatic colonized mothers to their infants in the uterus by aspiration of the infected amniotic fluid after premature membrane rupture and at the time of vaginal delivery (1). GBS carriage screening at weeks 35-37 of gestation and subsequent intrapartum antibiotic prophylaxis (IAP) for culture-positive women is recommended in many countries (4). Several studies have revealed that implementation of the IPA strategies has led to decline in the incidence of early onset GBS neonatal infections (4). Penicillin or ampicillin is the antibiotic of choice for prophylaxis and treatment of GBS infections. Other options include cefazolin for patients who do not exhibit immediate-type hypersensitivity to β-lactam antibiotics and clindamycin, erythromycin, or vancomycin for penicillin-allergic patients (5). Surveillance has shown that GBS remains universally susceptible to penicillins and glycopeptides such as vancomycin. However, *in vitro* studies indicate that the rate of killing of GBS by these antibiotics is relatively slow compared with other streptococcal pathogens (6). Recently, limited studies reported the elevated penicillin MIC level for group B streptococci (7–10). In several studies, significant resistance to clindamycin and erythromycin has been identified (11, 12).

Serotyping is an effective epidemiological tool for studying GBS. There are ten distinct GBS serotypes based on capsular polysaccharide antigen, namely Ia, Ib, II, III, IV, V, VI, VII, VIII and IX (13, 14). Capsular polysaccharide has been identified as a major virulence factor of GBS and consists of the main composition of GBS vaccines (15, 16). The antigenicity and immunogenicity of capsular antigens differs between serotypes and is associated with invasiveness of GBS diseases (17). The predominance of GBS serotypes changes over time and varies with geographical region and population (18).

Due to the growing concern regarding emerging antibiotic resistance for GBS and importance of serotype distribution of the GBS isolates, the objectives of this study were to determine GBS antimicrobial resistance pattern as well as GBS serotypes distribution in our population.

### Media and Chemicals

Todd-Hewitt broth and Muller-Hinton agar were purchased from sigma (St. Louis, MO, USA)and Merck (Merck, Darmstadt, Germany) companies respectively. Antibiotic disks for antimicrobial susceptibility testing were obtained from Hi-Media (Himedia Laboratories, Pvt. Ltd., Mumbai, India). The E-test strips for penicillin, ampicillin, erythromycin and clindamycin were obtained from BioMerieux, France. Vancomycin powder was purchased from Sigma company.

### GBS strains

Fifty six strains of GBS were used in this study. The strains were isolated from vaginal and rectal swabs collected between January 2008 and April 2008 from 420 pregnant women at 35-37 weeks of gestation attending to 11 clusters of obstetric clinic in Ardabil (19). The strains were stored in Todd-Hewitt broth along with 15% glycerol at -70°C until tested.

### Antimicrobial susceptibility

The MIC (Minimum inhibitory concentration) for clindamycin, penicillin, ampicillin, erythromycin and vancomycin against isolates were determined using E-test strips. Additionally, antimicrobial resistance pattern for ceftriaxone (100 µg), cefazolin (30 µg), ciprofloxacin (10 µg), and co-trimoxazole (25 µg) were determined using disk diffusion method by employing the antibiotic disks on Muller Hinton agar supplemented with 5% sheep blood. The clinical laboratory standard institute guidelines were used to interpret the results (CLSI 2010). Since zones of streptococcal growth inhibition to determine resistance are not available for cefazolin, we used values for Staphylococci.

### Serotyping

The strains were typed based on specific capsular antigen using standard antiserum (Strep B Latex Kit, Statens Serum Institute, Copenhagen, Denmark). The typing sera used in this study were Ia, Ib and II-VIII. Non-typeable isolates were designated NT.

### Statistical analysis

Data were analyzed using descriptive statistics.

## RESULTS

### Antimicrobial susceptibility pattern

The results of MIC tests showed all of isolates were susceptible to ampicillin, vancomycin and penicillin [MIC range 0.016-0.12 µg/mL, 0.094-0.75 µg/mL and 0.016-0.064 µg/mL respectively]. According to CLSI MIC values ≤ 0.25, ≤ 0.12 and ≤ 1 µg/mL are considered as susceptible for ampicillin, penicillin and vancomycin. Among the isolates 1 (1.7%) showed reduced susceptibility pattern to penicillin (MIC; 0.25 µg/mL) The overall MIC range of erythromycin and clindamycin was 0.016-0.5 µg/ml and 0.016-16 µg/ml respectively. There were 3 (5.3%) isolates semi sensitive (0.25-1 µg/ml) to erythromycin (2; 0.5 µg/ml and 1; 0.38 µg/ml) and 2 (3.5%) isolates to clindamycin (1; 0.5µg/ml, 1; 0.38 µg/ml). Additionally 2 (3.5%) isolates were resistant (MIC ≥ 1µg/ml) to clindamycin (1; 16 µg/ml, 1; 2 µg/ml).

According to disk diffusion test, 47 (83.9%), 8 (14.2%) and 7 (12.5%) isolates were resistant to Co-trimoxazole, ciprofloxacin and ceftriaxone respectively and 45 (80.3%), 44 (78.5%) and 1 (1.7%) isolates showed semi sensitive pattern to ceftriaxone, ciprofloxacin and cefazolin respectively (Table 2).

**Table 1:** Distribution of MIC for common antibiotics in GBS isolated from Iran (January – April, 2008).

MIC values µg/ml	Antimicrobial Agents				
	
	Penicillin n (%)	Ampicillin n (%)	Erythromycin n (%)	Clindamycin n (%)	Vancomycin n (%)
16	-	-	-	1 (1.78)	-
2	-	-	-	1 (1.78)	-
0.75	-	-	-	-	1(1.78)
0.5	-	-	2 (3.44)	1 (1.78)	4 (7.14)
0.38	-	-	1 (1.78)	1 (1.78)	11(19.64)
0.25	1(1.78)	-	1 (1.78)	2 (3.44)	38(67.85)
0.19	-	-	1 (1.78)	-	1(1.78)
0.125	-	4 (7.14)	-	-	-
0.094	-	23 (39.65)	-	2 (3.44)	1(1.78)
0.064	14 (25)	19 (32.75)	3 (5.36)	2 (3.44)	-
0.047	18 (32.14)	5 (8.6)	9 (16.07)	22 (39.28)	-
0.032	12 (21.42)	2 (3.44)	28 (50)	18 (32.14)	-
0.023	7 (12.5)	1 (1.72)	6 (10.67)	5 (8.92)	-
0.016	1 (1.78)	2 (3.44)	5 (8.92)	2 (3.44)	-
MIC Range	0.25-0.016	0.125-0.016	0.5-0.016	16-0.016	0.075-0.094

**Table 2:** Antibiotic susceptibility pattern of GBS isolates in Iran, (January – April, 2008).

Antibiotics	Susceptibility profile		
	
	Sensitive n (%)	Intermediate n(%)	Resistant n(%)
Penicillin^a^	56 (100)	-	-
Ampicillin^a^	56 (100)	-	-
Erythromycin^a^	53 (94.7)	3 (5.3)	-
Clindamycin^a^	52 (93)	2 (3.5)	2 (3.5)
Vancomycin^a^	56(100)	-	-
Cefazolin^b^	55 (98.2)	1 (1.78)-	-
Ceftriaxone^b^	4 (7.1)	45 (80.3)	7(12.5%)
Ciprofloxacin^b^	4 (7.1)	44 (78.5)	8 (14.2)
Co-trimoxazole^b^	8 (14.2)	1 (1.78)	47 (83.9)

a Susceptibility pattern was determined by E-test strips

b Susceptibility pattern was determined by disk diffusion method

### Serotypes distribution

Serotypes V (19.6%), II (12.5%) and IV (12.5%), were the most frequent followed by serotypes III (10/7%) and VI (10.7%), Ib (8.9%), Ia (7.1%), VII(5.3%) and VIII (5.3%); 7.1% of strains were nontypeable. Distribution of resistance-phenotypes among GBS serotypes is presented in Table 3.

**Table 3:** Distribution of resistance-phenotypes among GBS serotypes isolated from Iran (January – April, 2008).

Serotype ( n)	Resistance-phenotype	

	Resistant (%)	Intermediate (%)
Ia (4)	CO (100)	CI (100), CF (75)
Ib (5)	CO (100), CF (40), CI (20)	CI (80), CF (60)
II (7)	CO (85), CF (14), CI (14), CM (14.28)	CO (14), CF (85), CI (85), EM (14.28)
III (6)	CO (66), CF (33), CI (33), CM (16.66)	CF (40), CI (20), CZ (13)
IV (7)	CO (66)	CI (85), CF (85)
V (11)	CO (90), CF (9), CI (18),	CI (72), CF (81), EM (9.09), CM (18.18)
VI (6)	CO (100), CI (16)	CI (66), CF (100)
VII (3)	CI (66), CF (33)	CI (100), CF (66)
VIII (3)	CO (100)	CI (66), CF (66)
NT^a^ (4)	CO (75), CF (25)	CF (75), CI (50), EM (25)

Abbreviation: CO. Co-trimoxazole; CI. Ceftriaxone; CF. Ciprofloxacin; CM. Clindamycin; EM. Erythromycin; CZ. Cefazolin

a Non-typable

## DISCUSSION

The implementation of intrapartum prophylaxis with antibiotics to prevent perinatal GBS infections has reduced the incidence of GBS disease by 70% (4). However, the increased use of intrapartum chemoprophylaxis to prevent group B streptococcal disease has raised public health concerns over the emergence of antibiotic resistance among GBS strains and the possibility of increasing antibiotic resistance of other common pathogens affecting neonatal health (1). In this study all 56 strains were uniformly susceptible to penicillin and ampicillin. Similar findings have been reported for GBS strains around the world (20–24). One isolate showed elevated level of MIC against penicillin. Previously, similar findings had been reported against penicillin and ampicillin for GBS strains (7–10, 23) and penicillin treatment failure observed in practice frequently (25, 26). The emergence of strains with higher MIC levels to penicillin could indicate an emerging public health problem and suggests that susceptibility trends of these pathogens should be monitored carefully over the time.

In this study 96.7% and 93% of isolates were susceptible to erythromycin and clindamycin respectively. While antibiotic resistance varies by location and year of study conduction, the resistance to erythromycin and clindamycin are common in the GBS strains. It has been reported between 4 to 58.3% and 2.3% to 57.9% for erythromycin and clindamycin respectively in published literatures (11, 12, 21, 23, 27). However, the rate of resistance to erythromycin and clindamycin in this study were relatively low.

According to disk diffusion tests, a high proportion of the isolates were not susceptible (resistant or intermediate) to Co-trimoxazole (85.68%), ciprofloxacin (92.7%) and ceftriaxone (45%). High resistance to Co-trimoxazole has recently been described in GBS isolates (28). However, resistance to ciprofloxacin and ceftriaxone indicate a public health concern. Ceftriaxone is used for chemoprophylaxis in pregnant women during delivery in study setting. We detected one cfazolin-intermediate resistant GBS isolate which could indicate an emerging public health concern. Cefazolin is recommended for patients, who are at low risk for penicillin anaphylaxis (5).

Global serotyping distribution of a given GBS serotype varies according to geographical location, ethnic variation and time of study conductance (18). Our findings showed that the overall predominant GBS serotypes were serotypes V, II and IV representing about 44.6% of all serotypes. The seroprevalence of GBS at Ardabil is comparable to the study of Nahaee et al that have been conducted in other city in north west of Iran (29) but differs from seroprevalence of GBS reported from central Iran (23). These results show the ethnic variation of serotypes in pregnant women. It has been documented that subtle regional differences in demographic makeup can affect GBS serodistribution within the same country (21). GBS serotypes are associated with pathogen virulence (30) and somewhat with antimicrobial resistance pattern (11, 12). In this study over all, the resistance phenotype was similar between serotypes. Resistance to erythromycin, clindamycin and cefazolin was restricted to given serotypes. However, taking into account the small size of samples and low frequency of resistance to erythromycin, clindamycin and cefazolin determining the statistical relationship between resistance-phenotypes and GBS serotype was not possible. In conclusion, the results of this study provide an important vehicle for evaluating the public health risks posed by changes in distribution of GBS serotypes and choosing an appropriate vaccine composition and antimicrobial agents to prevent the GBS disease.

## Notes

**Funding:** This study was funded by the Vice-Chancellor for research at Ardabil University of Medical Sciences.

**Ethical approval:** Not required.

This work was partially presented at 5th International Conference on Infectious Diseases, Ditan, China.
